# Impulsivity and the Experience of Desire in the Choice of Erotic Stimuli

**DOI:** 10.3390/ijerph17144943

**Published:** 2020-07-09

**Authors:** Lara Salguero Lucas, Miguel Ángel Pérez Nieto, Silberio Sáez Sesma, Fernando Gordillo León

**Affiliations:** 1 Department of Psychology. Faculty of Education and Health. Camilo José Cela University, 28692 Madrid, Spain; mperez@ucjc.edu (M.Á.P.N.); fgordillo@ucjc.edu (F.G.L.); 2 Instituto de Sexología y Psicoterapia AMALTEA, 50007 Zaragoza, Spain; silberio@amaltea.org

**Keywords:** erotic desire, impulsivity, personality factors, behavioral trial

## Abstract

(1) Background: the relationship between erotic desire and personality factors is still relatively understudied. (2) Objective: to study the influence of the experience of desire, as well as impulsivity in the choice of videos, as the behavioral variable in the experimental trial. (2) Method: the sample consisted of 48 adult subjects, who took part in an experimental study that involved watching videos. (3) Results: the linear regression analysis revealed that the behavior involved in choosing videos is predicted by the sexual desire felt at the time of the trial, and not by stable personality factors, such as impulsivity or general self-report levels of sexual desire. (4) Conclusion: it is observed that the specific moment or situation and the behavior have a bigger impact on the erotic desire experienced at the time of the test than certain personality traits, as well as the previous and habitual levels of erotic desire of which an individual reports.

## 1. Introduction

There is currently no consensus on the definition of sexual or erotic desire across the different disciplines and sciences that study it. The definitions provided by Kaplan and Guastavino [1] who defined it as the motivational stage of the human sexual response (HSR), and Fuertes [2], who referred to erotic desire as a subjective emotional experience, are some of those that continue to inform professional practice in this field and the scientific study of that construct.

According to the measuring instrument used in it, this study is based on the understanding of desire furthered by Spector et al. [3], who contend that sexual desire refers to the interest a subject shows toward a sexual activity, with the qualification, what’s more, that such an activity is merely cognitive, and therefore measurable through the quantity and strength of the thought sensitive to sexual stimuli.

### 1.1. Conceptualization of Erotic Desire

Erotic desire has an important place in scientific research, and its studies are related to personal, social, relationship and/or health factors, gauging their importance in quality of life and sexual satisfaction in different contexts and situations. Studies addressing erotic desire among migrant sex workers [4], the sociological research relating sociocultural factors with erotic desire [5] and the medical research relating the endocrinological control of sexual desire in men and arousal/erection [6] highlight the range of scenarios present in the study of erotic desire. Interest in the field of clinical sexology and psychology focuses on, among other things, the relational factors involved in the activation or inhibition of desire [7] or on emotional disorders that may impact upon their development [8]. In this study, the measurement of erotic desire was considered using the Sexual Desire Inventory focused on three factors [9]. The authors throw out the idea of facing three types of desire: dyadic in couples, dyadic towards attractive people and solitary desire. From this perspective, we may understand there are several kinds of erotic desire, thereby rendering it inappropriate to measure a person’s desire from a general perspective without taking into account other situational variables that may be involved. As revealed by interactionist personality theories [10], an individual’s behavior is influenced by their environment, and not only by personality trait/mood factors. Understanding there are different types of erotic desire depending on different variables validates the notion of considering desire to be a complex construct influenced by the environment, and not only by personality factors. Staying with this notion, studies such as the one conducted by Prause et al. [11] in their investigation of an information processing model for understanding the variability in individuals’ level of erotic desire, contend that a new stimulus may be a decisive factor in the response to sexual stimuli, even affirming that the amount of attention captured by environmental stimuli is a major predictor of the level of erotic desire, even more so than the emotional responses that might be elicited by such stimuli. 

Erotic desire in a relationship and desire toward attractive individuals are clearly different, because, although they are factors that refer to dyadic desire, they are different concepts [9]. Other scholars in the field of sexology have already qualified certain difference in the type of female desire classifying this according to the object to which it was addressed. So, Cabello [12] talks about: an objectless desire that may be defined in physiological terms as an impulse; a desire toward a specific object or a given person, which in classical terms would involve romantic love: and a post-arousal desire typical of women in long and stable relationships, in which the activation of desire comes after the start of sexual relations.

This last type of desire is related to Basson’s model of female sexual response [13], in which the author contends that the general model of HSR propounded by Kaplan [14] is more closely related to women’s erotic desire at the start of a relationship, and not, therefore, to their desire in long relationships in which the SR, and therefore the workings of desire, change. Basson thus explains the importance of understanding that female sexual desire is different to the male one, depending on its start and continuation. Basson affirmed that women’s desire generally responds to a series of rewards or gains that do not have as much to do with the merely biological aspect as with the relational one, with this factor being one of the main motivational forces underpinning female desire. 

In this model, where male desire is more closely related to a biological impulse, and where rewards on an affective and/or relational level do not carry so much weight, and where female desire is generally fueled by some gain in terms of affection, Basson confirmed that greater commitment, greater affective ties and intimacy, an emotional engagement with a partner would be the main motivating factors triggering the cycle of sexual response on subsequent occasions. 

It seems clear that, although dyadic desire may be present in different situations, it is not always the same or expressed in the same manner. Which makes us think, in relation to the classification provided by Moyano et al. [9], it may sometimes be possible to record low levels of dyadic desire toward a stable partner and, by contrast, that this dyadic desire toward an attractive person may be very high. 

### 1.2. Erotic Desire and Personality Factors

There are few studies in the scientific literature that focus on the prediction of personality factors in the activation or inhibition of erotic desire. Making practical use of the prior, general and relatively stable levels of desire that individuals report in their interviews for informing an action protocol designed to improve the current situation may sometimes involve biased information, unless consideration is given to other situational factors that may have a bearing on the individual’s levels of desire. On the other hand, certain personality variables may be related to the appearance of erotic desire, and even predict it.

As expressed in Gray’s Theory of Personality [15], there are two neurobehavioral systems at the core of personality, and which regulate the sensitivity to punishment and reward [16].

Thus, based on this key notion, impulsivity would not be related so much to an irreflexive behavior or way of acting as to a motivation more focused toward, and sensitive to, signs of reward and/or relief [17].

Individuals’ results regarding sensitivity to reward can therefore be expected to go some way to predicting subsequent behavior in the experimental trial. As reported by Lankveld et al. [18], regarding risky sexual behaviors based on the Dual Control Model of Sexual Response, the Behavioral Inhibition and Activation Scale (BIS/BAS), based on Gray’s Theory of Personality [19], would be a good predictive instrument for these kinds of behavior. We therefore understand that a greater sensitivity toward reward could explain certain behaviors related, according to this theory, to extroversion. A focus on the relationship between impulsivity and sexual behavior reveals the strong relationship between this personality variable and impulsive behavior related to sex, where anxiety, sexual behavior and frequency are linked to a component of impulsivity [20]. Accordingly, Zuckerman’s Sensation Seeking Theory [21] posits a clear relationship between this trait and impulsivity, describing the disinhibition factor as an impulsive way of seeking sexual stimulation [17]. Some studies on the matter have revealed that people with more impulsive traits and with a greater tendency to seek sensations are those that behave in a sexually riskier manner [22,23]. This suggests that people with higher scores in sensation seeking are those that find it more difficult to inhibit certain behaviors they find arousing. By contrast, the study conducted by Jansen et al. [24] has found that taking sexual risks is not related to a propensity toward sexual inhibition. 

Understanding desire according to its biological and psychosocial nature as an emotion and a motivational phase of HSR would explain the relationship between it and certain personality dimensions that might have an influence on desire’s performance in situations that the individual finds appetizing or aversive.

This study sets out to analyze the possible relationship between the impulsivity variable and the experience of desire among the subjects in the sample, and its predictive power on behavior measures through an experimental trial. For this, it is proposed that the impulsiveness variable, measured through the SR scale, will predict a greater choice of videos with erotic charge. In turn, the subjects’ self-reported desire through SDI will also precede greater choice of erotic stimuli.

Finally, the desire experienced by the subjects during the test is expected to influence the choice of videos for the test: greater desire felt during the behavioral test, greater choice of videos with erotic charge.

## 2. Materials and Methods 

### 2.1. Participants

This sample has involved 48 subjects, of whom 38 are women (79% of the sample) and 10 are men (21% of the sample), with the youngest aged 18 and the oldest aged 62, with the sample recording an average age of 25.92 years. 

Out of all the subjects studied, 29 of them have a partner (60% of the sample), and 19 do not (40% of the sample). 

For men, the age range was between 19 and 62 (mean [*M*] = 27.40; and standard deviation [SD] = 13.17). For women, the age range was between 18 and 52 (mean [*M*] = 25.52; and standard deviation [*SD*] = 9.20). All the participants were Spanish. Subjects were recruited through a non-probability snowball sampling. There was no incentive for participants. Participation was completely voluntary.

The descriptive data of the sample for the levels of erotic desire of the SDI and Sensitivity to Punishment and Sensitivity to Reward (SCSR) will be indicated first, indicated in Table 1 and Table 2 respectively. The Sexual Desire Inventory indicates the mean and standard deviation of dyadic sexual desire (sum of dyadic desire towards the couple and towards attractive people), solitary sexual desire, dyadic sexual desire towards attractive people and dyadic sexual desire towards the couple. 

### 2.2. Instruments

Each subject is administered these tests in the following order:

#### 2.2.1. Sexual Desire Inventory (SDI)

This inventory is related to the definition of sexual desire provided by Spector et al. [3].

It is a version adapted to Spanish in 2006 by Ortega et al. [25]. The original version of the Sexual Desire Inventory was drafted by Spector et al. in 1996. 

The instrument in Spanish is a self-report consisting of 13 items. Factor 1 measures the dyadic sexual desire and that corresponds to items 1, 2, 3, 4, 5, 6, 7, 8 and 9. On the other hand, Factor 2 measures the solitary sexual desire and that corresponds to items 10, 11, 12 and 13. The internal consistency for the first factor is α = 0.87 and for the second α =0.88. In a later revision of the instrument, the possibility of being on a scale with three factors was raised, dividing dyadic desire into two types of desire: dyadic desire towards the couple and dyadic desire towards attractive people [9]. The factors would be dyadic desire towards a couple (items 1, 2, 3, 6, 7, 8 and 9), dyadic desire towards attractive (items 4 and 5) and solitary desire (items 10, 11, 12 and 13). The internal consistency of the SDI for our sample was α = 0.80. The internal consistency for the first factor (desire dyadic couples) is α = 0.79; for the second (desire dyadic attractive) is α = 0.84; and the last factor (desire solitary) is α = 0.90. As observed, the internal consistency of the SDI for our sample is similar to the data obtained in the validation of the scale. The internal consistency data shows a high consistency, close to 1, which implies a high reliability of the instrument in our sample.

#### 2.2.2. Sensitivity to Punishment and Sensitivity to Reward Questionnaire (SPSRQ)

This self-report questionnaire is related to the dimensions of anxiety and impulsivity in Gray’s theory. It involves a Spanish language version that was validated in 2001 by Torrubia et al. [26]. It consists of 48 items with dichotomous questions in which the subject is required to answer Yes or No. It consists of two subscales, each one of which has 24 items. The first subscale is related to Sensitivity to Reward, which in turn is related to the Behavioral Activation System (BAS) defined in Gray’s theory. This subscale is designed to measure those behaviors used to obtain reinforcements. On the other hand, the second subscale measures Sensitivity to Punishment, which is related to the Behavioral Inhibition System (BIS) in Gray’s Theory, measuring negative outcomes or punishments. The questionnaire’s internal consistency is α = 0.82 for Sensitivity to Reward, and α = 0.75 for the Sensitivity to Punishment scale. The internal consistency for our sample was α =.73 for Sensitivity to Reward, and α = 0.89 for the Sensitivity to Punishment scale. The internal consistency data of the SCSR for our sample, like the Sexual Desire Inventory, show values close to 1, which confirm a good reliability of the instrument in our study.

### 2.3. Procedure

**Figure 1:**
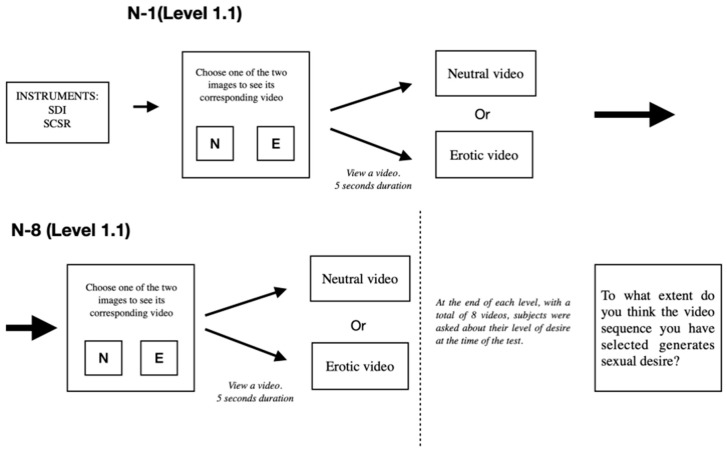
Description of the experimental task.

The following Figure 1 is a description of the experimental task:

Following the abovementioned tests, when the questionnaires were administered each subject individually underwent a behavioral trial, as defined forthwith:

A computer was used to show the subjects a series or images and videos. The trial was administered on an individual and solitary basis. The subjects were shown two images that were displayed on the same screen. The program used did not guarantee the randomization of the images, so it was decided to show the photographs with erotic content on one side of the screen, the left side, and images with neutral content on the right side of the screen. One image had an erotic content and the other had what we referred to as a neutral content. The images with neutral content referred to animals or plants. They had no sexual connotation. Beforehand, and using the chosen software, each image was assigned a video corresponding. Each image with neutral content and with erotic content, was a frame of the video that the subjects would see next once they had chosen the photo. When the two photographs were presented to them on the screen, the subjects had to choose one of them to start the corresponding video. If subjects chose an image with neutral content, they would watch a video with neutral content. On the other hand, if they chose an image with erotic content, they would see a video with erotic charge.

Participants had to choose a photo taking into account which video they wanted to view. The images did not appear with a certain time. The videos lasted 5 s.

The videos, and therefore the images, were classified by levels, with a total of four levels divided in turn into two levels each: (Level 1.1- Level 1.2); (Level 2.1- Level 2.2); (Level 3.1- Level 3.2); and (Level 4.1- Level 4.2). The videos with an erotic content corresponding to Level 1 were less explicit than, for example, the videos corresponding to Level 4. In the videos and images of the first levels, there were no genitalia or scenes with intercourse. The latest test videos do show genitalia and penetration and intercourse scenes.

The images and videos were not modified or pixelated. All images and videos were chosen and supervised by the study researchers. In order to carry out the statistical analyses, a numerical transfer was made. Each video chosen with neutral content was assigned the value (1), and each video with erotic content was assigned the numerical value (2). In this way, the minimum score for each level would be eight and the maximum sixteen. When the subjects watched eight videos in a row, they were asked a brief question: “To what extent do you think the video sequence you have selected generates sexual desire?”, to measure the degree of erotic desire perceived by four response options (no desire (0); little desire (1); really desire (2) and much desire (3)). In this way, the minimum score would be zero and the maximum would be three.

The subjects viewed a total of 128 images and 64 videos, choosing one video for each pair of images. Procedure described in Figure 1.

The administering of the questionnaires and the behavioral trial were held in a room where the subjects were on their own at all times. At the beginning of the trial, they were told what it involved, and they were reassured that no one would see or hear their results. 

All the subjects freely agreed to take part in the trial. Each subject took around 30 min to complete the task.

## 3. Results

Below is a descriptive analysis of the subjects’ choice of videos by levels and the levels of erotic desire they experienced at the time of the test.

To verify the existing relationship and the predictive capacity between the study variables measured through self-registration tests (erotic desire and impulsivity) and the behavioral test, correlation and linear regression analyzes were carried out using the SPSS version 25 program. The results are detailed below:

- Correlation analyses indicate that there is no relationship between the data obtained in the self-registration tests and the behavioral test. That is, there is no relationship between the general erotic desire found in the self-registration test and the behavior when faced with erotic stimuli, in this case the choice of videos. Along these same lines, there is also no correlation between lonely erotic desire and the choice of videos.

- A very weak and negative correlation is observed between the Reward Sensitivity scale and the choice of videos. (Indicated in Table 3). The regression analysis confirms a predictive power of the independent variable of 0.7%. (R^2^ = 0.007). These data do not allow us to determine that the levels of sensitivity to the reward can predict the behavior with erotic load of the test. 

- With regards to the variable choice of videos, its best prediction is explained by the variable desire felt in trial, with its coefficient of determination being R2 = 0.50. This means that the desire the subjects report they have felt during the trial and as a result of their behavior at this time is the one that will record the highest percentage when predicting the individual’s subsequent behavior. The effect size in this case is of high magnitude (f^2^ = 1.07). The results are indicated in Table 3 and Table 4. 

- Likewise, the data in Table 5 and Table 6 reflect that the two variables with the highest predictive power of the desire variable in the test are, on the one hand, the levels of solitary erotic desire and, on the other, with a lower predictive power, the levels of dyadic desire towards people. Attractive. After a more detailed analysis, it is observed that the independent variable solitary erotic desire explains 30.7% of the dependent variable, thus, indicating a high predictive power of felt desire in a test with erotic charge.

There is a significant correlation between the variable dyadic desire toward another person and the variable desire felt. Following a linear regression analysis with those variables, where the independent variable is dyadic desire toward an attractive person and the dependent variable is desire felt, it is noted that this independent variable explains 15.7% of the dependent variable. This means that experiencing higher levels of dyadic desire toward an attractive person in our study may predict 15.7% of the desire felt, as reported by the subjects when undertaking the experimental trial. The coefficient of determination in this case is R^2^ = 0.157.

## 4. Conclusions

The study’s findings reveal a very weak and negative relationship between the variable impulsivity and the choice of videos with erotic content in the behavioral trial. The results therefore show that more stable personality variables, such as impulsivity in this case, do not have a predictive power regarding behaviors in contexts with erotic charge. In this case, it would be necessary to replicate the study with a larger sample to better understand the relationship between sensitivity to reward (impulsivity) and the choice of videos. If we consider Gray’s Theory of Sensitivity toward Reinforcement [19], it confirms that sensitivity toward reward is related to behavioral activation in those behaviors designed to obtain reinforcements, such as sex, for example. Studies that focus on sexual behavior from a clinical perspective in relation to hypersexuality or the lack of control over impulses confirm the relationship between this personality variable and sexual behavior [27,28]. Contrary to what we expected, the results show that this personality variable will not have such an impact on the activation of behavior with an erotic charge. One possible explanation for these results is that impulsivity does not influence sexual behavior when this is more closely related to the viewing of videos or images with an erotic charge. In the line with the findings reported in the study conducted by Wetterneck et al. [29] factors related to impulsivity, such as seeking sensations or taking risks, correlate weakly with the frequency of the use of pornography. Given that behavior is measured here through videos with an erotic charge, it seems logical to understand that our personality variable does not have the expected influence on sexual behavior.

Another possible explanation for these results involves the approaches formulated through theories of personality trait/mood compared to situationist theories. Some studies on sexual behavior have reported that certain personality factors have a high correlation with hypersexuality, or behaviors, such as sexting [30,31]. By contrast, and in relation to the findings reported here, it may be understood that the environment and an individual’s experience of the situation may have more predictive power in certain behaviors with an erotic charge than their personality trait/mood factors. Along these lines, studies such as the ones conducted by Murnen, indicate that in terms of women’s unwanted sexual activity, situational variables are important for predicting their reactions [32]. In relation to this, in the present study there is a bias in the group, due to the high percentage of women that leads us to think that the situational variables in sexual activities become very important in this sex.

It is important to stress that there is a weak relationship between the variable solitary erotic desire and the choice of videos in the trial. Our data indicate that an individual’s reported levels of solitary desire are not necessarily related to subsequent sexual behavior with an erotic charge. In relation to this, and specifically with dyadic sexual behaviors, Basson confirms the existence of studies derived from different instances of research that stress that the motivation for sexual activity in a relationship may be different to solitary sexual desire [33]. Moreover, no relationship was found either between the variable choice of videos and dyadic erotic desire. This is related to the definitions of erotic desire provided thus far, as scholars, such as Spector et al. [3], have defined desire as the interest shown toward a sexual activity, which does not imply that there has to be sexual activity before or after. In other words, it is once again made clear that the self-report regarding an erotic behavior does not mean a greater predisposition toward such behavior. As Ortega et al. contend: “The Sexual Desire Inventory measures sexual desire as a mainly cognitive variable. This means that an interest in sexual behavior and effective sexual behavior are separate.” [25] (p. 148). 

In contrast to our results where the subjects’ self-reported erotic desire does not correlate with the behavioral test, choice of videos, and taking into account the high number of women in our study, the research findings of Conaglen y Evans conclude that the self-reported desire does not influence the response of men, but it does influence the response of women with less erotic desire, who rated sexual images as less pleasant and less exciting [34].

According to our results, therefore, the present situation and behavior has a bigger impact on the erotic desire experienced at that given moment than the prior and habitual levels of erotic desire the individual reports. We may assume accordingly that we will not be able to predict an individual’s desire in specific circumstances, based on the normal levels of desire they have recorded, but instead on the desire they experience at that moment. This is related to the conclusions reached in their study by Beck et al. [35], where the focus was placed on the experience of sexual desires among university students, concluding that the experience of sexual desire reflected in the results acted as a motivator for subsequent sexual behavior, either in a relationship or alone. In other words, it is once again noted that the experience of sexual desire has considerable power when predicting subsequent sexual behavior, making it clear, once again, that erotic desire and sexual behavior do not have to go hand-in-hand, or be similar constructs, as there may sometimes be sexual activities without any prior desire [13,35]; or the other way round, there may be an erotic desire with no subsequent sexual activity. As Zurbriggen and Yost have concluded, there are other motivations besides erotic desire and pleasure for prompting sexual behavior [36] and these two aforementioned components are not needed for a sexual activity to take place. This is reflected in women’s erotic desire where, as Parish and Hahn contend, they may be sufficiently motivated to experience sexual activity for reasons other than the desire to perform a sexual activity, per se [37].

This suggests that the notion of understanding an erotic desire as a purely cognitive variable without taking into account its affective nature may be a mistake, and therefore lead us to consider the affective and motivational component of erotic desire within a three-pronged model of the same [2] that advances the possibility of referring to an experience of desire with major predictive power regarding sexual behavior.

With regards to the variable referred to here as desire felt, a highlight among those levels of desire reported by the subjects during the behavioral trials, and as a result of the choice of videos, is the high prediction between that variable and the behavior prompted, thereby reflecting once again that the erotic desire an individual feels once an action has begun, and once desire has been triggered, carries more weight in the individual’s subsequent behavior than prior personality variables.

The little significance of stable personality variables such as impulsivity or general levels of erotic desire when predicting behavior in erotic contexts seems to indicate that in terms of sexual behaviors, the most stable personality traits will not be so important for understanding and explaining the subsequent behaviors manifested in erotic contexts.

With regards to the erotic desire toward attractive people, this is considered a dyadic desire, albeit with clear differences regarding dyadic desire in a relationship, with these being different concepts [9]. As seen in the present study, the dyadic desire toward a partner does not influence behavior with an erotic charge nor the subsequent behavior that such behavior may prompt, in contrast to dyadic behavior toward an attractive person, which does largely predict the desire felt. In this same vein, Marshall and Levy have already stressed the importance of physical attraction when taking part in sexual activities and when choosing a partner [38]. These findings induce us to consider the importance that the attraction of a person or situation may have in the activation of desire. As reflected in certain models of sexual therapy [12,39,40] for some time now fantasies and erotica have been used in clinical practice in specific situations and contexts to increase a subject’s low levels of desire.

Fantasies are a resource that is widely used by specialists in cases of low desire that pose problems in relationships. Prescribing the use of sexual thoughts through texts, videos or images is currently considered a good method for increasing desire, imbuing a specific situation with greater eroticism and attraction.

Although some studies have stressed that fantasies and sexual thoughts are not the same, as, unlike fantasies, the latter reveal the affect with which they are experienced [41] the two concepts are closely related to the sexual response, and specifically to erotic desire, as described in some studies [35,42].

There is therefore a plethora of studies reporting the relationship between fantasies and erotic desire [43,44]. Other studies on the matter address the relationship between desire and sexual pleasure and their influence on fantasies, even referring to the importance that prior sexual experiences have in the real world when explaining the desire and sexual pleasure expressed in fantasies [36].

It is important to consider these findings and stress the notion that anything we find attractive may be more readily imbued with eroticism and change an individual’s levels of erotic desire. 

All this prompts us to consider the importance of working with the appropriate variables when addressing situations and cases in which desire causes difficulties in a relationship. One of the aims in a relationship may be to work as a couple, providing a specific situation with greater eroticism in order to increase each individual’s attractiveness in their partner’s eyes. This is the stance taken by the intervention model proposed by McCarthy, who considers that the couple-based approach reinforces the concept of attraction, proposing, among other tasks, techniques that heighten the level of attraction between partners [45].

In clinical practice, increasing a couple’s level of mutual attraction, a change of setting, and heightened eroticism are all key aspects when addressing a dysfunction in levels of erotic desire, whereby making the couple and the situation more attractive may be a great help when increasing their levels of desire and even the predisposition to erotic behavior. It should be noted that one of the limitations of the study has to do with the instrument of measurement of erotic desire. The Inventory of Sexual Desire (SDI) focuses mainly on the measurement of desire based on masturbatory and coital behaviors, understanding this construct as an interest towards sexual activity, this being a cognitive activity, thus reducing the conceptualization of desire without taking into account other important factors such as the emotional component or the quality of external and internal inducers in their activation.

## Figures and Tables

**Table 1:** Descriptive statistics of the participant (*n* = 48). Sexual Desire Inventory (SDI).

Variables	M		SD	
	Women	Men	Women	Men
**Dyadic Desire**	45.26	49.70	11.34	9.62
**Solitary Desire**	14.57	18.00	8.45	5.16
**Dyadic Desire -Attractive-**	8.52	9.40	4.11	4.22
**Dyadic Desire -Couple-**	36.73	40.30	9.75	7.18

Note. M: mean; SD: standard deviation; Dyadic Desire: dyadic desire factor; Solitary Desire: solitary sexual desire factor; Dyadic Desire Attractive: dyadic desire factor towards attractive person; Dyadic Desire Couple: dyadic desire factor in couples.

**Table 2:** Descriptive statistics of the participant (*n*= 48). Sensitivity to Punishment and Sensitivity to Reward Questionnaire (SPSRQ).

Variables	M		SD	
	Women	Men	Women	Men
**SC**	9.53	8.70	6.52	5.01
**SR**	10.10	11.40	4.45	2.59

Note. M: mean; SD: standard deviation; *SR*: scale sensitivity to reward; *SC*: scale sensitivity to punishment.

**Table 3:** Linear regression analysis results.

Dependent Variable	Predictor Variable	R^2^	Adjusted R^2^	Standard Error of the Regression	Durbin-Watson (Statistic)
Choice of Videos	Sensitivity to reward	0.007	−0.014	20.392	2.156
	Desire Felt in Trial	0.517	0.506	14.227	2.349

**Table 4:** Linear regression analysis results (Standardized coefficients).

Dependent Variable	Predictor Variable	Unstandardized	Beta	T	P	f^2^
Choice of Videos	Sensitivity to reward	−0.417	−0.085	−0.582	0.564	0.007
	Desire Felt in Trial	2079	0.719	7.015	<0.001	1.07

**Table 5:** Linear regression analysis results.

Dependent Variable	Predictor Variable	R^2^	Adjusted R^2^	Standard Error of the Regression	Durbin-Watson (Statistic)
Desire Felt in Trial	Solitary Erotic Desire	0.307	0.292	5.895	1.858
	Dyadic Desire toward an Attractive Person	0.175	0.157	6.430	2.087

**Table 6:** Linear regression analysis results (Standardized coefficients).

Dependent Variable	Predictor Variable	Unstandardized	Beta	T	P	f^2^
Desire Felt in Trial	Solitary Erotic Desire	0.487	0.554	4.510	<0.001	0.44
	Dyadic Desire toward an Attractive Person	0.713	0.418	3.122	0.003	0.21
